# Determinants of Burden and Satisfaction in Informal Caregivers: Two Sides of the Same Coin? The CUIDAR-SE Study

**DOI:** 10.3390/ijerph16224378

**Published:** 2019-11-09

**Authors:** Leticia García-Mochón, Luz María Peña-Longobardo, María del Río-Lozano, Juan Oliva-Moreno, Isabel Larrañaga-Padilla, María del Mar García-Calvente

**Affiliations:** 1 Escuela Andaluza de Salud Pública (EASP), 18080 Granada, Spain; leticia.garcia.easp@juntadeandalucia.es (L.G.-M.); mariadelmar.garcia.easp@juntadeandalucia.es (M.d.M.G.-C.); 2 Instituto de Investigación Biosanitaria de Granada ibs.GRANADA, 18012 Granada, Spain; 3 CIBER en Epidemiología y Salud Pública (CIBERESP), 28029 Madrid, Spain; 4 Departamento de Análisis Económico y Seminario de Investigación en Economía y Salud (SIES), Universidad de Castilla-La mancha, 45071 Toledo, Spain; LuzMaria.Pena@uclm.es (L.M.P.-L.); Juan.OlivaMoreno@uclm.es (J.O.-M.); 5 Departamento de Salud del Gobierno Vasco, 20010 San Sebastián (Gipuzkoa), Spain; ilarranaga@euskadi.eus

**Keywords:** informal care, caregiver, burden, satisfaction, health related quality of life, gender

## Abstract

The aim of this study conducted in Spain was to analyze and compare burden, severe burden, and satisfaction among informal caregivers in relation to health-related quality of life (HRQoL), type and duration of caregiving, perceived social support, and use of social and health care services. We performed multivariate analyses to identify variables associated with caregiver burden, severe burden, and satisfaction with caregiving, stratified by gender. The results showed that secondary or third-level education, performance of ungratifying tasks, negative coping with caregiving, and more years providing care were associated with greater burden. Variables with protective effect were better perceived health of the person being cared for, better caregiver HRQoL, and high perceived social support. Women were 75% more likely to experience severe burden compared with male caregivers. Burden was reduced by high perceived social support in the case of women and by high caregiver HRQoL in the case of men. The main determinant of caregiving satisfaction for both men and women was perceived social support (OR = 3.11 and OR = 6.64). This study shows the need for interventions that promote gender equality and social support as a means of relieving burden and severe burden and improving satisfaction in both male and female caregivers.

## 1. Introduction

As the population ages and people with chronic diseases are living longer, a growing dependence on care is likely to result in an increasing shift from professional to informal care. This situation places an even greater burden on informal caregivers and maintaining support from the public sector in a secondary position [1].

Informal care is the unpaid care to cover the basic needs of people with limited autonomy, provided by family, friends, or neighbors [2]. Due to its heterogeneous nature, informal care has no clear boundaries, and definitions vary among authors and studies and are strongly influenced by cultural, historical, and social factors. Informal care is not considered a profession in its own right, as it does not come with employment rights, nor as such work schedules or rest periods, and while caregivers might receive a family allowance or other types of subsidies, their work is generally unpaid. The characteristics of informal care, together with the contexts in which it is delivered and the extent to which it is taken for granted, are largely responsible for its invisibility and little social recognition. It is unpaid work based on an emotional attachment between two people that occurs within the confines of the home, and additionally, it is perceived as belonging to women and is undervalued both socially and economically [3,4].

Caregiving can have positive, negative, or both positive and negative effects on caregivers [5]. The demands of caring for a dependent person can cause considerable burden, negatively affecting mental and physical health. Caregivers are more likely to experience feelings of depression, loss of control and autonomy [6], anxiety, and guilt [6,7] and to have a worse quality of life [8] and more health problems [9]. They also incur considerable opportunity costs, such as time lost that could be spent doing other activities such as paid work, fulfilling family obligations, social life, and leisure [10]. Caregiving burden, whether objective or subjective, is a multidimensional construct that is influenced by numerous factors. Hours spent providing care [11,12], the state of health and level of dependence of the person being cared for [12,13,14,15], financial difficulties, and poor access to social and health care services [11,16,17] have all been linked to greater burden. The evidence is not so clear, however, when these factors are analyzed by gender. Although much research appears to indicate that caregiving has a greater negative impact on women than on men, other studies have not found any differences. These discrepancies, however, are probably due to the use of different methodologies and uncertainly about which variables influence gender differences and to what extent [18].

Whilst most studies tend to focus on the negative aspects of caregiving, there is some evidence that caregiving can be a positive experience, particularly in terms of psychosocial effects related to personal well-being and satisfaction with caring for another person [19,20]. Lopez et al. [21] found that greater levels of satisfaction among caregivers were associated with a better previous care-recipient relationship, choosing to become a caregiver, keeping free time for oneself, less need for venting emotions, and not working outside the home. Very little, however, is known about the determinants of caregiver satisfaction or about how these differ between men and women. In the few studies that have performed gender-stratified analyses, the results have been contradictory, with some authors reporting that men derive greater satisfaction than women from caregiving [21,22,23] and others finding non-significant differences [17,24,25,26].

The aim of this study was to investigate determinants of caregiver burden, overburden, and satisfaction associated with caregiving. More specifically, it aims at the identification and analysis of differences between men and women in relation to these three aspects and associated factors such as caregiver quality of life, caregiving characteristics, perceived social support, and use of social and health care services. We also wished to analyze whether the determinants of burden are similar or different from those associated with satisfaction.

## 2. Materials and Methods

This was a cross-sectional study carried out in 2013 in an adult caregiver population in two Spanish provinces: Granada (Andalusia) and Gipuzkoa (Basque Country). It was part of the first wave of studies conducted within the multicenter longitudinal CUIDAR-SE project analyzing the health-related quality of life (HRQoL) of male and female caregivers in these two provinces.

The study population was made up of people aged 18 or more living in a family home who provided informal care to a dependent person living in the same or another home and who were registered as caregivers with the Primary Health Care District of Granada or the Social Services of the Provincial Council of Gipuzkoa.

The caregivers selected to participate in the study were identified using a three-stage cluster random sampling approach in which municipalities were established as primary units, census sectors within these municipalities as secondary units, and caregivers as final units. Municipalities were stratified by size and caregivers by gender.

Data were collected between September and December 2013. Face-to-face interviews were held with the caregivers using an ad hoc structured questionnaire [27] designed on the basis of previous research in the field, services and interventions targeting caregivers in the study areas, and validated scales and instruments used in Spain to analyze caregiver health and quality of life [28]. The questionnaire includes items to assess the two dependent variables in our study: caregiver burden and satisfaction with caregiving role. Other items covered, and used as independent variables, were caregiver characteristics (gender, age, place of residence, household income—adjusted by household size and composition by OECD modified scale, HRQoL—measure through EQ5D-5L proxy questionnaire, and negative coping with caregiving—classified as 1 if the caregiver refused to believe that caregiving was happening and 0 otherwise); health of the care recipient as perceived by the caregiver; characteristics of caregiving (years providing care and performance of ungratifying personal care tasks, defined in our study as having to change diapers); perceived social support and use of social and health care services (e.g., allowances, day centers, nursing homes, telecare, home care, nursing services, and support and training). Perceived social support was measured using the DUKE-UNC-11 [29], an 11-item questionnaire that assesses confidant support (having someone you can share thoughts with) and emotional support (demonstrations of love, affection, and empathy).

Caregiving burden was assessed using the Zarit scale, which is a 22-item questionnaire designed to assess how caregivers feel while providing care [30]. For each item, the caregiver is asked to indicate how often they feel each of the dimensions on a 5-point scale that consists of 1 (never), 2 (rarely), 3 (sometimes), 4 (quite frequently), and 5 (nearly always). The total possible score therefore ranges from 22 to 110 points. Scores of 22–47 indicate no or low burden, 47–55 moderate burden, and 56–110 severe burden.

To analyze caregiver satisfaction with their caregiving role, we used a specific questionnaire designed for the CUIDAR-SE study that analyzes five dimensions of satisfaction related to caring for someone [27]: (1) whether or not the caregiver feels closer to the care recipient as a result of looking after them (closeness), (2) whether they enjoy spending time with this person (enjoyment), (3) whether they feel their self-esteem has improved as a result of caregiving (self-esteem), (4) whether the pleasant moments experienced by the person being cared for brings the caregiver pleasure as well (empathy), and (5) whether caring for this person brings greater meaning to the caregiver’s life (added meaning to life).

The questionnaire was previously piloted among 20 participants, in order to guarantee its understanding. This pilot test concluded that the questionnaire was well understood and that, therefore, it was not necessary to modify any question. It was administered during a personal interview at the caregiver’s home. All caregivers selected to participate in the study received a letter from the Granada or Basque health authorities inviting them to participate in the study and explaining the objectives of the study and all the ethical and confidentiality aspects associated with their participation.

The project was approved by the Biomedical Research Ethics Committee of Andalusia and the Provincial Council of Gipuzkoa granted approval for accessing the necessary databases and registries.

Two types of statistical analyses were performed to analyze caregiver burden based on Zarit scores. In the first analysis, given the normal distribution of scores, the scale was used in its continuous form, with scores analyzed in an ordinary least squares model where Zarit score = β_0_ + β_1_ (middle-older age) + β_2_ (older age) + β_3_ (female) + β_4_ (primary studies) + β_5_ (secondary/third-level education) + β_6_ (Granada) + β_6_ (high HRQoL) + β_7_ (years of care) + β_8_ (years of care2) + β_9_ (high perceived social support) + β_10_ (performance of ungratifying tasks) + β_11_ (middle adjusted household income) + β_12_ (high adjusted household income) + β_13_ (coping with caregiving) + β_14_ (perceived care recipient health) + u_t_.

Adjusted high household income was denoted as 1 for a monthly income of over €1500 and as 0 otherwise. Middle adjusted household income was denoted as 1 for a monthly income of between €1000 and €1500. Older age was denoted as 1 for an age of older than 65 years and 0 otherwise. Middle-older age was denoted as 1 for an age of between 50 and 65 years. High HRQoL was classified as a score of over 0.85. Perceived social support was classified as high “1” for a Duke-UNE score of between 11 and >32. Finally, perceived care recipient health was denoted as 1 when the caregivers reported excellent or good health and 0 when they reported fair, poor, or very poor health.

In the second analysis, we analyzed the likelihood of severe caregiver burden using a dichotomous classification of Zarit scores: 1 for a score of 55 or higher and 0 for a lower score. Logit models were developed using the same explanatory variables as those used in the previous case. 

To analyze satisfaction with caregiving, we developed several logit models where the dependent variables were assigned a value of 1 for each of the dimensions considered if the caregiver was satisfied and 0 if not. The model used in each case was as follows: satisfaction/dissatisfaction with care (for each dimension) = β_0_ + β_1_ (middle-older age) + β_2_ (older age) + β_3_ (female) + β_4_ (primary studies) + β_5_ (secondary/third-level education) + β_6_ (Granada) + β_7_ (high HRQoL) + β_8_ (perceived care recipient health) + β_9_ (high perceived social support) + β_10_ (performance of ungratifying tasks) + β_11_ (middle adjusted household income) + β_12_ (high adjusted household income) + β_13_ (receipt of allowances) + β_14_ ( use of home social and health care services) + β_15_ (use of social and health care services outside the home) + β_16_ (use of other formal services) + u_t_.

Both the caregiver burden and satisfaction analyses were also stratified by gender.

## 3. Results

We analyzed 610 caregivers. Their main sociodemographic characteristics are shown in Table 1. Mean ± SD caregiver age was 59.82 ±14.47 years and 56% of the caregivers were women; 40.07% of caregivers had not completed primary education. The mean HRQoL score for the overall group was 0.82 ± 0.194 and coping with caregiving was identified in 4.92% of caregivers. Over 85% of care recipients received social and health care services at home, 17.38% received services outside their home, and 79.51% received allowances. The mean ± SD Zarit score was 49.69 ± 14.82. In terms of satisfaction with their caregiving role, 87.87% of caregivers felt closer to the person they were caring for, 84.92% enjoyed spending time with the person, 75.25% felt that their self-esteem had been boosted, 92.95% felt empathy, and 79.84% thought that caring for this person added meaning to their life. Several significant differences were detected between male and female caregivers. Men were older, had a higher household income, and in general felt more satisfied with their caregiving role, whilst women had been providing care for longer, received more allowances, and perceived stronger support from their social networks.

The strongest determinants of caregiver burden were gender, level of education, perceived health of the person being cared for, caregiver HRQoL, performance of ungratifying tasks, coping with caregiving, high perceived social support, and number of years providing care (Table 2). Caregiver burden was higher in women, who on average scored 3.64 points more on the Zarit scale than men, in caregivers with a secondary or third-level education (+5.44 points), in caregivers with negative coping with caregiving (+6.20 points), and in caregivers who had to perform ungratifying tasks (+8.82 points). By contrast, burden was lower in caregivers caring for a person they perceived to be in good or very good health (on average they scored 4.36 less on the Zarit scale) and caregivers who perceived having strong social support (−10.56 points) (Table 2). 

In the analysis of caregiver burden by gender, level of education, caregiver HRQoL, performance of ungratifying tasks, perceived health of care recipient, high perceived social support, and avoidance coping retained their significance as determinants of burden (Table 2). The quantitative effects, however, were stronger for men, as male caregivers with a high HRQoL scored on average 11.34 points less on the Zarit scale (vs. 6.87 points less for women). Likewise, men who performed ungratifying tasks scored 3.94 points more (vs. 3.07 points more for women), while those who used avoidance coping scored 6.77 points more (vs. 5.57 for women). The effects, however, were lower for perceived social support, with male caregivers with high perceived support scoring 8.82 points less on the Zarit scale compared with 12.09 points less for women.

The variables associated with severe caregiving burden (Zarit score ≥55 points) are shown in Table 3. Severe burden was more likely in women (75% higher probability than in men) and in caregivers with a high household income (OR = 2.85). Caregivers with a high HRQoL were 75% less likely to be severely burdened, while those who performed ungratifying tasks were 93% more likely. Likewise, caring for a person with good or very good health and feeling one has strong social support were associated with a 44% and a 76% lower probability of being severely burdened, respectively. On analyzing these results by gender, the quantitative effect for male versus female caregivers was higher for HRQoL and lower for social support. Additionally, in the case of male caregivers, having a high household income and using avoidance coping strategies were not significantly associated with severe burden. However, the performance of ungratifying tasks was associated with a higher probability of severe burden among men (OR = 2.54) but not women (non-significant effect).

The likelihood of severe caregiver burden was greater in women (+75%), caregivers with a high household income (OR = 2.85), and caregivers who had to perform ungratifying tasks (93%). By contrast, it was lower in caregivers with a high HRQoL (+75%), caregivers looking after a person deemed to be in good or very good health (44%), and caregivers who perceived a high level of social support (76%).

The main determinants of caregiver satisfaction with their role were high perceived social support (for all the satisfaction dimensions analyzed) and place of residence (for the dimensions of closeness, enjoyment, and empathy) (Table 4). The higher the level of perceived social support, the more likely it was that caregivers would be satisfied, particularly for the dimensions of enjoyment (OR = 3.51) and empathy (OR = 3.11). Furthermore, caregivers living in Granada were on average more likely to be satisfied than those living in Gipuzkoa and this was particularly evident in the empathy dimension (OR = 3.33).

On analyzing the determinants of caregiver satisfaction by gender (Table 5 and Table 6), both male and female caregivers were more likely to be satisfied when they perceived high social support (OR = 3.11 and OR = 6.64, respectively). Place of residence did not explain satisfaction in the case of female caregivers. Finally, female caregivers looking after a person who received social and health care services outside the home were less likely to feel satisfied with their caregiving role, particularly in terms of closeness (OR = 0.34).

## 4. Discussion

We have shown that caregiver burden and satisfaction are related constructs but have separate meanings and interpretations. Caregivers who expressed greater satisfaction with their caregiving role had lower caregiver burden scores on the Zarit scale for all the satisfaction dimensions. Whilst we found differences between determinants of burden and overburden and satisfaction, we also found that social support protected against burden and severe burden and favored greater satisfaction with care.

This study adds to the limited body of evidence on differences between male and female caregivers in terms of burden, overburden, satisfaction, and associated factors. The greater levels of burden and severe burden observed in the female caregivers in our series are consistent with reports from other studies that have evaluated burden experienced by caregivers looking after people with different health problems and levels of dependence [13,18]. The perception of greater burden in women may also be due to the little social recognition that women receive in return for their work. Social norms dictate that women should look after people in need and when a man takes on this role, he tends to gain more respect and admiration than women in the same position. Moreover, several studies have shown that female caregivers are more confined to the home and receive less support than men [31,32]. This is linked to strong gender norms that lead women to adopt and internalize the caregiver role much more strongly than men and they also tend to internalize the resulting burden, leading to more serious effects on their health and other aspects of life [4].

Higher income and education were also associated with greater burden in our series following adjustment for social support and other variables. This could be related to opportunity costs in terms of lost time and earnings [27,33] and with the psychological and emotional dimensions of burden. In many cases, having to divide one’s time between caregiving responsibilities, paid employment, and pursuit of leisure activities [5,34,35] places an additional burden on caregivers, causing greater emotional stress. Female caregivers and caregivers reaching retirement age, in worse health, and who dedicate more time to caregiving have been found to experience more negative employment impacts [5].

A negative emotional coping and having to perform ungratifying tasks (defined in our study as having to change diapers) were also associated with higher burden scores, regardless of perceived social support, suggesting, as reported elsewhere [36,37], that caregiving might not be considered so stressful if caregivers had access to psychological resources to help them cope with the burden of care or help with tasks that cause more stress. Interventions aimed at reducing amount of care may not be sufficient to alleviate feelings of burden if psychological, social, and material support is missing [38]. We also observed differences between male and female caregivers. Performing ungratifying tasks appeared to affect men more than women, while strong social support exerted a protective effect in women. This may be because men have not been socialized to be caregivers in the same way as women have, and when they adopt this role, the assumption of ungrateful tasks entails a greater perception of burden. In this respect, they have been found to delegate more arduous caregiving tasks before their health becomes seriously affected [4]. Interventions to mitigate these effects should consider these differences and care services should take a differentiated approach to help alleviate burden depending on the caregiver’s gender and type of care provided [32].

Better perceived care recipient health, and high perceived social support all acted as buffers against burden. These findings are consistent with previous reports that have shown a direct link between subjective burden and physical and mental health [16,39], care recipient health [13,14,15], and social support [17]. There is evidence that the age of the care recipient and the medical diagnosis also influence the burden. A recent article concludes that patients with dementia and depression are those that generate the greatest burden on their caregivers [40]. Some studies have shown that spouse caregivers of dementia report more burden than adult child caregivers [41,42]. A recent Spanish study attributed the highest subjective burden to women caring for their husbands. This could be due to differences in the perception of the caregiving situation, such as more perceptions of the over-responsibility or less positive perception by the wives [43].

As demonstrated in other studies, high social support is a key determinant of greater satisfaction with one’s role as a caregiver for both men and women [38,44,45,46]. Other people can help reduce stress levels by offering solutions, downplaying importance, or acting as a distraction [47]. As shown by Del Río et al. [8], male and female caregivers receive different types of support: while women seek less support than men and draw more on relatives than on formal services, men opt more for financial support, home help, and other forms of instrumental help. There is evidence that the structure and cohesion of caregiver networks are important aspects of social support exchanges and have an important impact on burden [48,49] and satisfaction [49]. The findings of our study highlight the need to promote social support interventions aimed at preventing or alleviating burden and overburden. Nevertheless, studies that have evaluated the effectiveness of such interventions have shown heterogeneous results [50].

A recent study from the CUIDAR-SE project [51] showed slight differences in the composition and structure of the personal networks of male and female caregivers. Men had wider and more diverse networks than women and also received more support from people outside their family circles, such as work colleagues and paid professionals. Women’s networks, by contrast, were less diverse and were composed mainly of women with similar sociodemographic profiles and often from the same family. These differences again suggest that it may be necessary to design different support interventions for male and female caregivers.

The findings of the current study show that women derived less satisfaction from their caregiving role than men in terms of enjoyment, self-esteem, and a meaningful life. It is important to note that the men in our series were mainly looking after a wife living at home. Kang et al. [33] found that caring for a spouse caused greater anxiety due to the intensive nature of this role. They also found, however, that spousal caregivers felt more fulfilled and socially accepted. The lower satisfaction results observed for women in these dimensions, however, could also be explained by the different caregiving roles that women and men adopt, with women internalizing a role they are expected to fulfil and men feeling more that they are acting altruistically. 

For women, home help services had a positive influence on satisfaction while external help services had a negative influence [8]. These effects were not observed in men. Having a higher education was also associated with lower levels of satisfaction in women and could be related to the greater effect observed for higher-level education on burden due to opportunity costs.

One possible limitation of our study is that we only analyzed caregivers registered as such with the health authorities. It is possible, however, that caregivers who have no contact with health or social services provide less intensive care. We believe, therefore, that the findings of our study can be extrapolated to caregivers providing similarly high levels of care to those in our series.

## 5. Conclusions

In conclusion, the findings of this study show that caregiver burden and satisfaction are influenced by several factors and that there are differences between men and women. Our results also indicate that social policies aimed at improving caregiver well-being should not be based on isolated measures, but rather take a holistic approach incorporating fiscal, social welfare, regulatory, and labor measures together with social service policies aimed at promoting training and strengthening social support networks. To this end, greater public investment is needed in dependent care assistance programs and caregiver support policies aimed at ensuring gender equality at all stages. Obviously, these policies should target the family environment and society as a whole.

## Figures and Tables

**Table 1:** Distribution of caregiver characteristics by gender.

	Total (*n* = 610)	Male (*n* = 265)	Female (*n* = 345)	Comparison of Means *p*-Value ^1^
**Caregiver characteristics**				
Female sex, (%)	56.56	-	-	-
Age, mean ±SD	59.82 ± 14.47	62.28 ± 16.28	57.94 ± 12.62	0.022 **
Province (Granada), (%)	51.31	49.8	52.2	0.63
Household income, mean ± SD	1157.59 ± 539.99	1212.51 ± 547.04	1113.87 ± 531.15	0.032 **
**Education**				0.1841
No studies completed, (%)	40.07	45.08	36.23	
Primary education, (%)	25.94	21.21	29.57	
Secondary/third-level education, (%)	33.99	33.71	34.20	
HRQoL, mean ± SD	0.827 ± 0.19	0.836 ± 0.20	0.821 ± 0.18	0.32
Coping with caregiving (%)	4.92	4.9	4.9	0.99
**Support received**				
**Social and health care services**				
In the home, (%)	85.74	84.15	86.95	0.33
Outside the home, (%)	17.38	13.96	20.00	0.051 *
Allowances, (%)	79.51	73.93	83.76	0.003 ***
Other services, (%)	66.56	66.03	66.95	0.81
Social support, (%)	80.16	76.98	82.60	0.08 *
Burden; Zarit score, mean ± SD	49.69 ± 14.82	46.94(14.29)	51.81(14.90)	
**Satisfaction with care**				
Overall satisfaction with care, (%)	87.70	88.30	87.24	0.69
Feeling close to care recipient, (%)	87.87	90.20	86.10	0.12
Enjoyment from spending time with care recipient, (%)	84.92	89.40	81.40	0.006 ***
Increased self-esteem as a result of caregiving, (%)	75.25	80.82	71.01	0.005 ***
Empathy (pleasure derived from seeing care recipient experiencing pleasure), (%)	92.95	95.10	91.30	0.070 *
Greater meaning to caregiver’s life as a result of caregiving, (%)	79.84	86.01	75.10	0.0008 ***
**Characteristic of care recipient**				
Perceived health by caregiver (excellent or good), (%)	64.85	67.04	63.18	0.32
**Caregiving characteristics**				
Years providing care, mean ± SD	9.40 ± 8.54	7.97 ± 7.35	10.49 ± 9.21	0.0004 ***
Performance of ungratifying tasks, (%)	51.14	48.30	53.33	0.22

^1^ Significance level: * Significant at 90%, ** Significant at 95%, *** Significant at 99%.

**Table 2:** Variables associated with informal caregiver burden (Zarit score): multivariate analysis differentiated by gender ^1^.

	All Caregivers (Zarit Score)		Male Caregivers		Female Caregivers	
	Marginal Effect ± SD	*p*-Value	Marginal Effect ± SD	*p*-Value	Marginal Effect ± SD	*p*-Value
**Caregiver characteristics**						
Female	**3.64 ± 1.14**	**0.002**	---------	---------	---------	---------
Secondary/third-level education	**5.44 ± 1.55**	**0.001**	**5.92 ± 2.54**	**0.021**	**5.55 ± 2.04**	**0.007**
Health-related quality of life (high) ^2^	**−8.82 ± 1.14**	**0.000**	**−11.34 ± 1.77**	**0.000**	**−6.87 ± 1.55**	**0.000**
Avoidance coping	**6.21 ± 1.91**	**0.001**	**6.77 ± 3.33**	**0.043**	**5.57 ± 2.17**	**0.011**
Perceived social support (high)	**−10.56 ± 1.44**	**0.000**	**−8.92 ± 2.10**	**0.000**	**−12.09 ± 2.00**	**0.000**
**Caregiving characteristics**						
Years providing care	**0.39 ± 0.19**	**0.041**	0.40 ± 0.32	0.208	0.43 ± 0.24	0.078
Performance of ungratifying tasks	**3.41 ± 1.06**	**0.001**	**3.94 ± 1.58**	**0.013**	**3.07 ± 1.50**	**0.042**
**Care recipient characteristics**						
Perceived health by caregiver	**−4.36 ± 1.15**	**0.000**	**−4.54 ± 1.67**	**0.007**	**−4.69 ± 1.65**	**0.005**
*n*	529		223		296	
F-Snedecor	19.69		9.04		11.52	
R-squared	0.3365		0.3766		0.2957	

^1^*p* < 0.05 was regarded as significant; significant *p*-values are marked in bold. Non-significant explanatory variables included in the model (omitted in the table): years of care squared, age, primary education, adjusted household income. ^2^ EQ-5D score ≥0.85.

**Table 3:** Variables associated with informal caregiver intensive burden (Zarit score): multivariate analysis differentiated by gender ^1^.

	All Caregivers (Zarit Score)		Male Caregivers		Female Caregivers	
	Odds Ratio	*p*-Value	Odds Ratio	*p*-Value	Odds Ratio	*p*-Value
**Caregiver characteristics**						
Age >65 years	0.52	0.060	**0.28**	**0.033**	0.85	0.730
Female	**1.75**	**0.015**	---------	---------	---------	---------
Secondary/third-level education	**2.05**	**0.018**	1.85	0.259	**2.61**	**0.014**
Adjusted household income (middle)	**1.70**	**0.039**	2.09	0.080	1.40	0.319
Adjusted household income (high)	**2.85**	**0.001**	2.18	0.148	**3.50**	**0.002**
Health-related quality of life (high) ^2^	**0.25**	**0.000**	**0.16**	**0.000**	**0.31**	**0.000**
Coping with caregiving						
Perceived social support (high)	**0.24**	**0.000**	**0.32**	**0.005**	**0.17**	**0.000**
**Care recipient characteristics**						
Perceived health by caregiver	**0.56**	**0.019**	0.69	0.36	**0.43**	**0.011**
Performance of ungratifying tasks	**1.93**	**0.003**	**2.54**	**0.009**	1.49	0.174
*n*	529		233		296	
LR chi2	141.10		60.86		86.35	
Pseudo R2	0.2076		0.2221		0.2174	

^1^*p* < 0.05 was regarded as significant; significant *p*-values are marked in bold. Non-significant explanatory variables included in the model (omitted in the table): years of care, years of care squared, age, primary education. ^2^ EQ-5D score ≥0.85.

**Table 4:** Variables associated with informal caregiver satisfaction with caregiving role: multivariate analysis for all caregivers ^1^.

	Satisfaction with Care (General)		Satisfaction with Care (Closeness)		Satisfaction with Care (Enjoyment)		Satisfaction with Care (Self-Esteem)		Satisfaction with Care (Empathy)		Satisfaction with Care (Added Meaning to Life)	
	Odds Ratio	*p*-Value	Odds Ratio	*p*-Value	Odds Ratio	*p*-Value	Odds Ratio	*p*-Value	Odds Ratio	*p*-Value	Odds Ratio	*p*-Value
**Caregiver characteristics**												
Age >65 years	**-**	**-**	**-**	**-**	**-**	**-**	**2.21**	**0.017**	**-**	**-**	**2.53**	**0.009**
Female	**-**	**-**	**-**	**-**	**0.43**	**0.003**	**0.61**	**0.030**	**-**	**-**	**0.48**	**0.003**
Secondary/third-level education	**-**	**-**	**-**	**-**	**-**	**-**	**0.48**	**0.019**	**-**	**-**	**0.51**	**0.043**
Granada	**2.15**	**0.035**	**2.15**	**0.008**	**-**	**-**	**-**			**0.016**	**-**	**-**
Health-related quality of life (high) ^2^	**-**	**-**	**-**	**-**	**2.43**	**0.005**	**-**	**-**	**-**	**-**	**-**	**-**
Perceived social support (high)	**4.39**	**0.000**	**2.38**	**0.008**	**3.51**	**0.000**	**2.94**	**0.000**	**3.11**	**0.005**	**2.57**	**0.000**
Social and health care services outside the home	**0.52**	**0.049**	**0.47**	**0.024**	**-**	**-**	**-**	**-**	**-**	**-**	**-**	**-**
*n*	550		550		550		550		550		550	
LR chi2	39.69		30.47		50.15		65.89		24.98		62.18	

^1^*p* < 0.05 was regarded as significant; significant *p*-values are marked in bold. Non-significant explanatory variables included in the model (omitted in the table): age (middle), primary education, adjusted household income, ungratifying tasks, perceived care recipient health, allowances, social and health care services in the home, other social services. ^2^ EQ-5D score ≥0.85.

**Table 5:** Variables associated with informal caregiver satisfaction with caregiving role: multivariate analysis for men ^1^.

	Satisfaction with Care (General)		Satisfaction with Care (Closeness)		Satisfaction with Care (Enjoyment)		Satisfaction with Care (Self-Esteem)		Satisfaction with Care (Empathy)		Satisfaction with Care (Added Meaning to Life)	
	Odds Ratio	*p*-Value	Odds Ratio	*p*-Value	Odds Ratio	*p*-Value	Odds Ratio	*p*-Value	Odds Ratio	*p*-Value	Odds Ratio	*p*-Value
**Caregiver characteristics**												
Age >65 years	**-**	**-**	**5.17**	**0.036**	**-**	**-**	**4.60**	**0.011**	**-**	**-**	**-**	**-**
Granada	**5.63**	**0.008**	**8.87**	**0.002**	**-**	**-**	**-**	**-**	**9.15**	**0.021**	**-**	**-**
High health-related quality of life ^2^	**3.11**	**0.034**	**-**	**-**	**-**	**-**	**-**	**-**	**5.54**	**0.024**	**-**	**-**
High perceived social support	**3.11**	**0.027**	**-**	**-**	**3.96**	**0.005**	**3.04**	**0.007**	**-**	**-**	**2.84**	**0.018**
*n*	243		243		243		243		243		243	
LR chi2	28.05		22.04		24.25		41.15		14.42		24.01	
Pseudo R2	0.1578		0.1368		0.1430		0.1684		0.1421		0.1178	

^1^*p* < 0.05 was regarded as significant; significant *p*-values are marked in bold. Other explanatory variables not significant included in the model (omitted in the table): age (medium), primary education, adjusted household income, ungratifying tasks, perceived care recipient health, allowances, social services in the home, social and health care services outside the home, other social services. ^2^ EQ-5D score ≥0.85.

**Table 6:** Variables associated with informal caregiver satisfaction with caregiving role: multivariate analysis for women ^1^.

	Satisfaction with Care (General)		Satisfaction with Care (Closeness)		Satisfaction with Care (Enjoyment)		Satisfaction with Care (Self-Esteem)		Satisfaction with Care (Empathy)		Satisfaction with Care (Added Meaning to Life)	
	Odds Ratio	*p-*Value	Odds Ratio	*p*-Value	Odds Ratio	*p*-Value	Odds Ratio	*p*-Value	Odds Ratio	*p*-Value	Odds Ratio	*p*-Value
**Caregiver characteristics**												
Secondary/third-level education	**-**	**-**	**-**	**-**	**-**	**-**	**0.42**	**0.027**	**-**	**-**	**0.44**	**0.043**
Perceived social support (high)	**6.64**	**0.000**	**2.57**	**0.030**	**3.46**	**0.001**	**2.78**	**0.003**	**5.10**	**0.002**	**2.52**	**0.010**
Social and health care services in the home	**-**	**-**	**2.62**	**0.048**	**-**	**-**	**-**	**-**	**-**	**-**	**-**	**-**
Social and health care services outside the home	**0.42**	**0.037**	**0.34**	**0.008**	**-**	**-**	**0.50**	**0.039**	**-**	**-**	**0.48**	**0.033**
**Care recipient characteristics**												
Perceived health by caregiver	**-**	**-**	**-**	**-**	**3.04**	**0.007**	**-**	**-**	**2.97**	**0.046**	**-**	**-**
*n*	307		307		307		307		307		307	
LR chi2	30.72		21.59		32.47		35.16		21.64		41.56	
Pseudo R2	0.1293		0.0881		0.1081		0.0951		0.1215		0.1194	

^1^*p* < 0.05 was regarded as significant; significant *p*-values are marked in bold. Non-significant explanatory variables included in the model (omitted in the table): age, primary education, place of residence, adjusted household income, ungratifying, allowances, other social and health care services.
